# Evaluation of TLR Agonists as Potential Mucosal Adjuvants for HIV gp140 and Tetanus Toxoid in Mice

**DOI:** 10.1371/journal.pone.0050529

**Published:** 2012-12-13

**Authors:** Viviana Buffa, Katja Klein, Lucia Fischetti, Robin J. Shattock

**Affiliations:** Clinical Sciences, St. George's University of London, London, United Kingdom; Shanghai Medical College, Fudan University, China

## Abstract

In the present study we investigate the impact of a range of TLR ligands and chitosan as potential adjuvants for different routes of mucosal immunisation (sublingual (SL), intranasal (IN), intravaginal (IVag) and a parenteral route (subcutaneous (SC)) in the murine model. We assess their ability to enhance antibody responses to HIV-1 CN54gp140 (gp140) and Tetanus toxoid (TT) in systemic and vaginal compartments. A number of trends were observed by route of administration. For non-adjuvanted antigen, SC>SL>IN immunisation with respect to systemic IgG responses, where endpoint titres were greater for TT than for gp140. In general, co-administration with adjuvants increased specific IgG responses where IN = SC>SL, while in the vaginal compartment IN>SL>SC for specific IgA. In contrast, for systemic and mucosal IgA responses to antigen alone SL>IN = SC. A number of adjuvants increased specific systemic IgA responses where in general IN>SL>SC immunisation, while for mucosal responses IN = SL>SC. In contrast, direct intravaginal immunisation failed to induce any detectable systemic or mucosal responses to gp140 even in the presence of adjuvant. However, significant systemic IgG responses to TT were induced by intravaginal immunisation with or without adjuvant, and detectable mucosal responses IgG and IgA were observed when TT was administered with FSL-1 or Poly I∶C. Interestingly some TLRs displayed differential activity dependent upon the route of administration. MPLA (TLR4) suppressed systemic responses to SL immunisation while enhancing responses to IN or SC immunisation. CpG B enhanced SL and IN responses, while having little or no impact on SC immunisation. These data demonstrate important route, antigen and adjuvant effects that need to be considered in the design of mucosal vaccine strategies.

## Introduction

The development of a protective vaccine against HIV/AIDS represents the best hope to contain the spread of HIV-1 infection. Given that sexual transmission of HIV-1 is the predominant mode of HIV acquisition in adults [1], a key element for a successful preventive vaccine may be the ability to generate potent immune responses at the mucosal portals of entry (genital tract and rectum). The presence of specific antibodies at the portals of infection provides a first line of adaptive defence for the host against horizontal transmission and the induction of neutralizing or inhibitory anti-Env antibody responses is likely to be the primary component of an effective HIV vaccine [2]. Mucosal vaccination is considered an important strategy to induce local immune responses [3],[4] and different approaches, using DNA, viral vectors and protein based vaccines alone or in combination, are currently under investigation [5]. However given the potential compartmentalization of the mucosal immune system, selection of the most appropriate route of immunisation may be critical for the design of a successful preventive HIV vaccine. Indeed, mucosal responses appear to be more easily elicited by administering vaccines on mucosal surfaces than by parenteral immunisation [6],[7],[8]. Safety is also of paramount importance in vaccine design and, in this light, proteins are generally considered safe but often lack potency in eliciting immune responses when administered mucosally alone [7]. This likely reflects: the presence of local degrading enzymes; lack of penetration or uptake across mucosal barriers and lack of requisite danger signals required to trigger adaptive immunity.

For these reasons, adjuvants are thought to be particularly important for mucosal immunisation approaches in order to induce long lasting protective immunity. Different classes of compounds are currently under investigation as vaccine adjuvants [9] and, among these, Toll-like receptor (TLR) ligands represent very interesting candidates [10]. The TLRs are pathogen recognition receptors (PRR), present on different cell types, which are involved in the recognition of specific microbial molecular motifs. On binding to their respective ligands, TLRs mediate intracellular signalling pathways that lead to the production of pro-inflammatory cytokines, up-regulation of MHC molecules and amplification of B and T cell responses [11]. In this way, engagement of TLRs link innate and adaptive immune responses and can be exploited for adjuvanticity purposes. Many TLR ligands have proven to be very effective in augmenting both cellular and humoral immune responses in various models [11] and some ligands have been reported to be effective at enhancing systemic and local immune responses when administered intra-nasally [12],[13],[14]. Moreover, they were recently shown to be able to confer better mucosal protection in a SIV challenge model in macaques [15].

Several TLR ligands are currently being developed as adjuvants for human use. In particular, TLR4 ligand MPLA is licensed for human use in HPV and hepatitis B vaccines and TLR9 ligand, CpG-B, has been extensively tested in vaccine trials for hepatitis B and anthrax, where it was shown to be able to enhance specific antibody responses. Moreover, other ligands such as Pam_3_CSK_4_ (TLR2) and R848 (TLR7/8) are under investigation and proven to be safe in different clinical trials [16].

In this study we have evaluated the potential of several TLR ligands as adjuvants for mucosal immunisations in mice via three different routes of mucosal administration: intranasal (IN), intravaginal (IVag), sublingual (SL); and a parenteral route, subcutaneous (SC), as a control. We compared the responses induced against CN54gp140, a recombinant clade C envelope protein [17], versus those against the potent immunogen Tetanus toxoid (TT). In our study we also included chitosan, a polysaccharide widely used in vaccine formulations that can enhance immune responses, as control adjuvant [18].

Our approach focused on the evaluation of candidate adjuvants' ability to induce specific genital and systemic humoral responses, both IgG and IgA through different mucosal routes of immunisation. Moreover, IgG subclasses, IgG2a and IgG1, were investigated in order to address the influence of adjuvant and route of administration on the balance between Th1 and Th2-type immune responses.

## Materials and Methods

### Reagents

Tetanus toxoid was obtained from Statens Serum Institute and CN54gp140 was obtained from Polymun Scientific. The TLR ligands FSL-1 (TLR2/6), Poly I∶C (TLR3), Pam_3_CSK_4_ (TLR1/2), R848 (TLR7/8) were purchased from Invivogen, monophosphoryl Lipid A (MPLA, TLR4) from SIGMA and CpGB (TLR9) from MWG. Chitosan was provided by Novamatrix.

### Mice and immunisations

Ethics Statement: All animals were handled and procedures performed in strict accordance with the terms of a project licence (PPL 70/6613) granted under the UK Home Office Animals (Scientific Procedures) Act 1986 and the study was approved by the animal ethics committee of St. George's University of London. Mice were maintained in conditions conforming to UK Home Office guidelines to ameliorate suffering and were euthanized by cervical dislocation.

Female BALB/c mice, aged 6–8 weeks were purchased from Harlan. For vaginal immunisation protocols, prior to the first immunisation mice were given subcutaneously 2 mg of medroxyprogesterone acetate (Pharmacia Limited). Nasal and vaginal immunisations were performed in a final volume of 20 µl containing 10 µg of antigen (either gp140 or Tetanus Toxoid) and either 20 µg of TLR ligand or 100 µg of chitosan, in PBS. Sublingual immunisations were performed using the same amount of antigen and ligand in a final volume of 10 µl and, after each immunisation, animals were kept under anaesthesia with their head positioned in ante-flexion for 10 min to avoid swallowing. For the parenteral route, mice were immunised subcutaneously with the same amounts of antigen (10 µg) and adjuvant (20 µg for TLR ligands and 100 µg for chitosan) in a final volume of 50 µl. All the animals were vaccinated three times with an interval of two weeks in between immunisations. Blood samples were collected two weeks after the last immunisation by tail vein puncture and vaginal washes were collected, under anaesthesia, flushing the mouse vagina with 75 µl of PBS. For all immunisations and vaginal sampling mice were anaesthetised using Isoflurane-Vet (Merial).

### Mouse samples

Sera were collected 2 hours after bleeding, spinning the blood samples for 10 min at 23,000 g and collecting clear supernatants. Vaginal washes were treated with a protease inhibitor cocktail (SIGMA) for 30 min at 4°C then spun for 10 min at 23,000 g to remove cell debris. All samples were stored at −80°C.

### Detection of specific IgG and IgA

Serum and vaginal samples were tested for the presence of specific (gp140 or Tetanus toxoid) IgG and IgA using an in-house ELISA protocol. Plates were coated with 5 µg/ml antigen overnight at 4°C and blocked for 1 hour at 37°C in PBS containing 1% BSA (SIGMA). Samples were diluted in assay buffer (PBS containing 1% BSA and 0.05% Tween 20) and incubated for 1 hour at 37°C. Specific IgG was detected using a goat anti-mouse HRP (Serotec) antibody whilst IgA was detected indirectly using a goat anti-mouse biotin antibody (SouthernBiotec) and then adding streptavidin (R&D). Plates were read at 450 nm after addition of SureBlue TMB substrate (KPL) followed by 1N H_2_SO_4_ to stop the colorimetric reaction. Endpoint titres were calculated using GraphPad Prism version 4 as the reciprocal of the highest dilution giving an absorbance value equal or higher to the background (naïve mouse serum) plus two standard deviations. Cut-off value was set at 0.1.

### Detection of IgG subtypes

Specific IgG subclasses were detected as described above, using anti-mouse IgG1 HRP and anti-mouse IgG2a HRP (Serotec).

### Statistical analysis

The statistical difference between groups was determined by Mann-Whitney test and one way ANOVA. All analyses were performed using GraphPad Prism v 4.

Significant differences between the different antigen/adjuvant groups and the no adjuvant control group were indicated as follows: * for p≤0.05, ** for p≤0.01 and *** for p≤0.001.

## Results

In order to determine the impact of the route of immunisation on systemic and vaginal humoral responses to gp140, animals were immunised by sublingual, nasal, vaginal and parenteral routes with a range of TLR ligands (FSL-1 (TLR2/6), poly I∶C (TLR3), MPLA (TLR4), CpG-B (TLR9), Pam_3_CSK_4_ (TLR1/2), R848 (TLR7/8)) and chitosan. To evaluate the influence of the antigen on the responses to mucosal immunisation parallel experiments were performed using Tetanus Toxoid (TT).

### Sublingual immunisation with gp140 and TT

**Figure 1:**
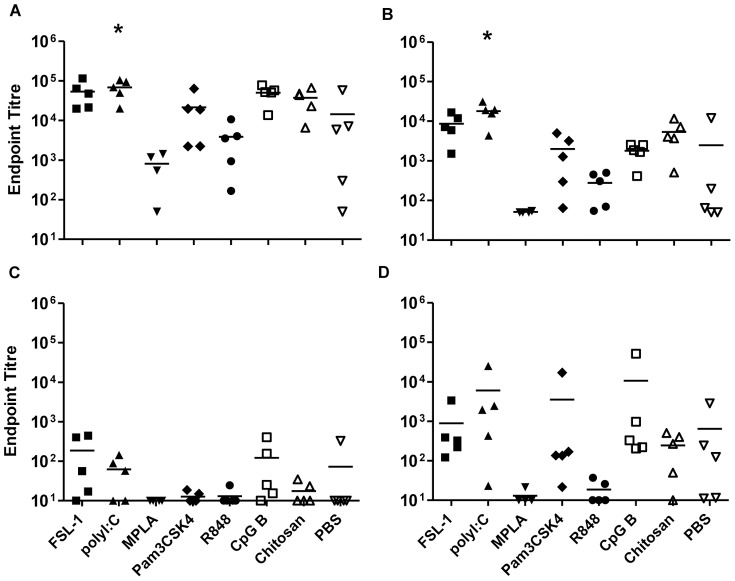
Sublingual immunisation with gp140. Endpoint titres for IgG (A, C) and IgA (B, D) in sera (upper panels) and vaginal washes (lower panels) from animals immunised three times with gp140 sublingually. Asterisks indicate significant differences between the different adjuvant/antigen groups and the PBS control group.

Sublingual immunisation with CN54gp140 induced good systemic IgG responses, with endpoint titres up to 10^5^ when the antigen was administered alone. A similar pattern in IgG and IgA responses was observed when the antigen was given in combination with FSL-1, Pam_3_CSK_4_, R848 or chitosan, whilst poly I∶C significantly increased systemic IgG and IgA titres (p = 0.03 and p = 0.015 respectively). MPLA was the only adjuvant candidate that appeared to dampen specific responses (Figure 1A and B). In vaginal wash samples, low but detectable IgG responses were observed in some animals (Figure 1C), however these were inconsistent with none of the groups showing detectable responses in all animals. In contrast, IgA titres were detected in all animals where antigen was administered with FSL-1, poly I∶C, Pam_3_CSK_4_ or CpG B. (Figure 1C and D).

IgG subclass analysis was performed to determine specific IgG1∶IgG2a ratios as a surrogate of Th1/Th2 biasing of systemic humoral responses. When gp140 was administered alone the IgG1/IgG2a ratio was 11 suggesting a Th2-biased response (Figure S1A). This trend was maintained for all adjuvants and appeared to be enhanced with Poly I∶C, R848 and chitosan, although not statistically different to gp140 alone.

**Figure 2:**
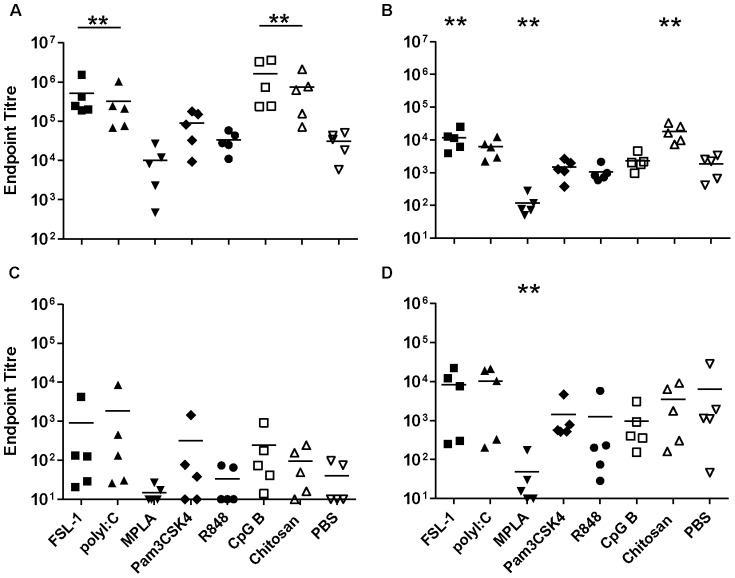
Sublingual immunisation with Tetanus toxoid. Endpoint titres for IgG (A, C) and IgA (B, D) in sera (upper panels) and vaginal washes (lower panels) from animals immunised three times with Tetanus toxoid sublingually. Asterisks indicate significant differences between the different adjuvant/antigen groups and the PBS control group.

To determine the impact of the antigen on specific responses induced by sublingual immunisation, parallel experiments were performed using Tetanus toxoid (TT). TT induced strong humoral systemic responses (mean specific IgG endpoint titre of 3×10^4^) when used alone. Significant increases in specific IgG above antigen alone were seen when TT was administered with FSL-1, Poly I∶C, CpG B or chitosan (p = 0.007), while an increase in specific IgA was seen for FSL-1 and chitosan (p = 0.007) (Figure 2A and B). In contrast, co-administration of TT with MPLA significantly decreased systemic IgA responses (p = 0.008). TT administered alone induced poor or undetectable vaginal IgG responses (Figure 2C), however FSL-1, Poly I∶C, and CpG B induced detectable vaginal IgG responses in all animals within each group, although these were still low. However, specific vaginal IgA responses were detectable for all animals receiving TT alone and these responses were similar to those seen with all adjuvants, with the exception of MPLA, which reduced titres of specific vaginal IgA (p = 0.015) (Figure 2C and D).

Specific IgG subclass analysis demonstrated that TT when given alone induced a balanced systemic IgG1/IgG2a ratio of 0.9 (Figure S1B) and this was maintained with all adjuvants except chitosan which gave a significantly increased IgG1/IgG2a ratio relative to TT alone.

### Nasal immunisation with gp140 and TT

**Figure 3:**
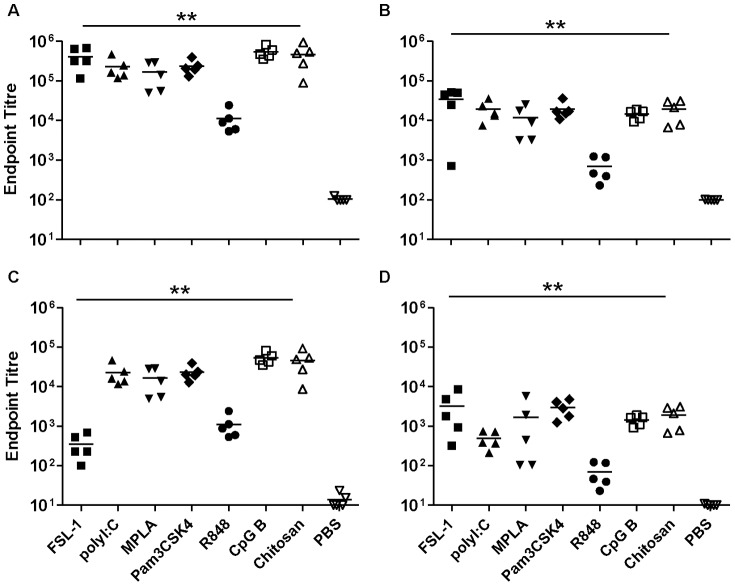
Intranasal immunisation with gp140. Endpoint titres for IgG (A, C) and IgA (B, D) in sera (upper panels) and vaginal washes (lower panels) from animals immunised three times with gp140 intranasally. Asterisks indicate significant differences between the different adjuvant/antigen groups and the PBS control group.

The administration of gp140 alone via the nasal route induced barely detectable systemic or local IgG and IgA responses. However, all adjuvant candidates tested promoted strong systemic IgG production, giving titres up to 5.33×10^5^ (p<0.01) (Figure 3A). Likewise, specific serum IgA titres were induced by all adjuvant candidates with serum titres of up to 3.4×10^4^. These were significant for all adjuvants (Figure 3B), however the effect of R848 was significantly lower than that of the other adjuvants for both IgG and IgA (p = 0.01). In vaginal wash samples, all adjuvants significantly increased specific IgG titres (p<0.01), which were below or at the cut-off for detection when gp140 was given alone. FSL-1 and R848 also augmented vaginal IgG responses but to a lesser extent (Figure 3C). For specific IgA, all the candidates significantly increased vaginal antibody titres but the enhancement mediated by R848 was significantly lower than that of the other adjuvants (Figure 3D).

IgG subclass analysis indicated that all candidates tested significantly increased both specific IgG1 and, with the exception of chitosan, IgG2a antibody titres (p<0.01) (data not shown). gp140 when administered alone gave an IgG1/IgG2a ratio of 3.5. FSL-1, MPLA, Pam_3_CSK_4_ and chitosan increased IgG1/IgG2a ratios promoting a Th2 biasing of responses that were significant for Pam_3_CSK_4_ and Chitosan. Conversely, poly I∶C and CpG-B lowered mean IgG1/IgG2a ratios although this did not reach a level of statistical significance (Figure S2A).

**Figure 4:**
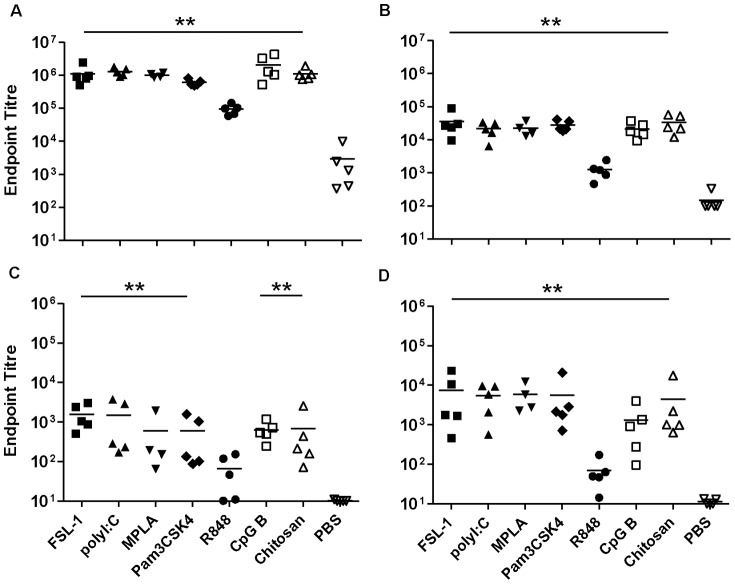
Intranasal immunisation with Tetanus toxoid. Endpoint titres for IgG (A, C) and IgA (B, D) in sera (upper panels) and vaginal washes (lower panels) from animals immunised three times with Tetanus toxoid intranasally. Asterisks indicate significant differences between the different adjuvant/antigen groups and the PBS control group.

When TT was given alone intra-nasally, appreciable systemic IgG responses were elicited, with an average titre of 2×10^3^. All the adjuvants significantly increased specific IgG and IgA titres in sera (p<0.01), up to an IgG titre of 2×10^6^ for CpG-B and an IgA titre of 3.6×10^4^ for FSL-1. The effect mediated by R848 was again significantly lower than that of the other candidates (Fig. 4A and B). For the vaginal antibody responses, TT in combination with the different adjuvants elicited good IgG titres, significantly higher than the group with TT alone (p<0.01) where titres were very low or undetectable. The only exception was the group with R848 that, although showing a positive trend, did not reach statistical significance (Figure 4C). Higher titres were also observed for specific vaginal IgA with all adjuvant candidates showing significantly increased titres (Fig. 4D), with a maximum average titre of 7.4×10^3^ for FSL-1

IgG subclass analysis of sera indicated that all candidates significantly increased IgG2a and IgG1 titres (data not shown) relative to antigen alone. The administration of TT alone intra-nasally gave an IgG1/IgG2a ratio of 20 suggesting a Th2 biasing of responses. This ratio was significantly increased when the antigen was used in combination with chitosan (Figure S2B), whilst poly I∶C, Pam_3_CSK_4_, R848 and CpG-B gave lower average ratios although differences were not statistically significant.

### Vaginal immunisation with gp140 and TT

**Figure 5:**
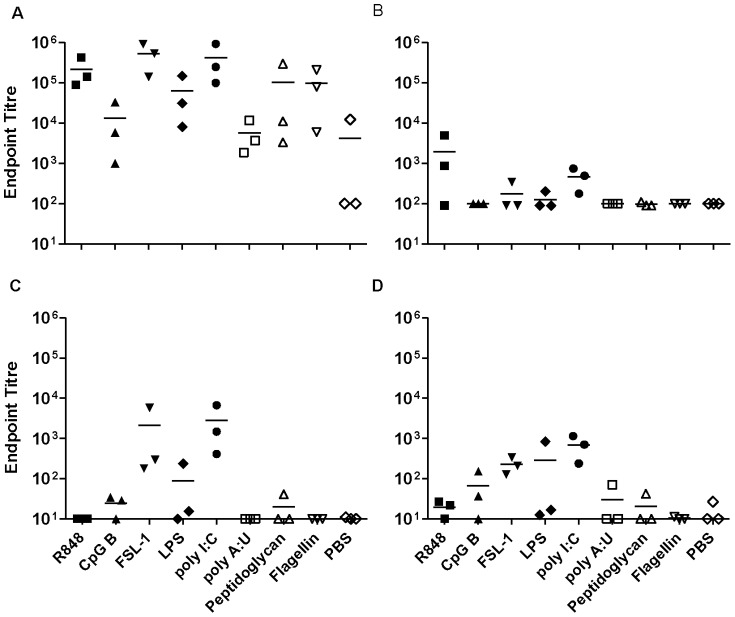
Intravaginal immunisation with Tetanus toxoid. Endpoint titres for IgG (A, C) and IgA (B, D) in sera (upper panels) and vaginal washes (lower panels) from animals immunised three times with Tetanus toxoid intravaginally.

Vaginal administration of CN54gp140 failed to induce detectable systemic or vaginal IgG and IgA responses. Likewise, none of the candidate adjuvants tested induced specific systemic antibody titres following vaginal immunisation. Lack of local vaginal responsiveness to gp140 was also observed for both IgG and IgA in all the groups tested (data not shown). In contrast, when TT was administered via the same route in a pilot experiment, the antigen alone gave low but detectable systemic IgG responses, with an average titre of 4×10^3^. Furthermore some TLR ligands such as FSL-1, poly I∶C, LPS and R848 increased systemic IgG titres up to a maximum value of 10^6^ for FSL-1 (Figure 5A). Systemic IgA titres on the other hand were low or not detectable (Figure 5B). In the genital mucosal compartment, both FSL-1 and poly I∶C increased specific IgG and IgA titres (Figure 5C and D). However, the TT specific antibody titres observed were overall lower than those obtained with the other routes of immunisation.

### Parenteral immunisation with gp140 and TT

**Figure 6:**
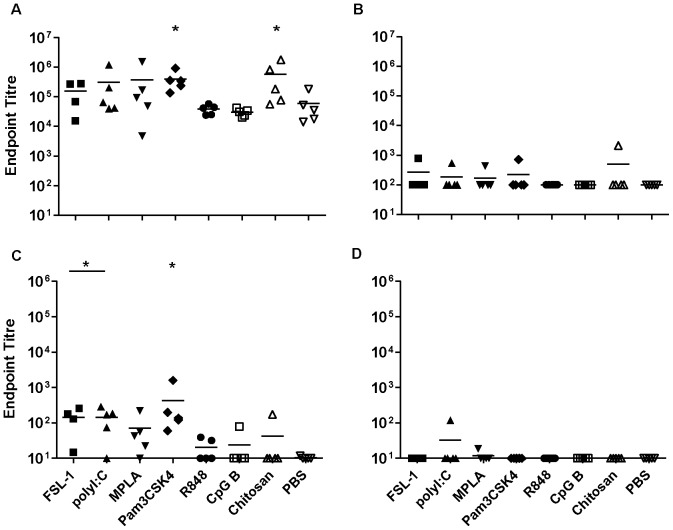
Subcutaneous immunisation with gp140. Endpoint titres for IgG (A, C) and IgA (B, D) in sera (upper panels) and vaginal washes (lower panels) from animals immunised three times with gp140 subcutaneously. Asterisks indicate significant differences between the different adjuvant/antigen groups and the PBS control group.

To compare mucosal and parenteral immunisation routes, gp140 and TT administered by the subcutaneous route. gp140 alone induced very strong systemic IgG responses, with an average titre of 6.0×10^4^. Of the adjuvants tested, Pam_3_CSK_4_ and chitosan significantly enhanced antibody titres up to 10 fold (p = 0.016 and 0.03 respectively) (Figure 6A). Conversely, specific serum IgA responses were barely above background in the antigen-alone group and none of the adjuvants increased specific IgA titres. A similar pattern was observed in vaginal wash samples, with detectable IgG titres and very poor or no specific IgA responses. FSL-1, poly I∶C and Pam_3_CSK_4_ significantly increased mucosal IgG titres giving titres up to 4.2×10^2^ (p<0.01) (Fig. 6C).

IgG subclass analysis of sera, indicated that gp140 alone induced a very high average IgG1/IgG2a ratio of above 50 (Figure S3A) that was similar to responses induced in the presence of chitosan. In contrast all the TLR adjuvant candidates significantly reduced this ratio providing a more balanced response, most evident with MPLA.

**Figure 7:**
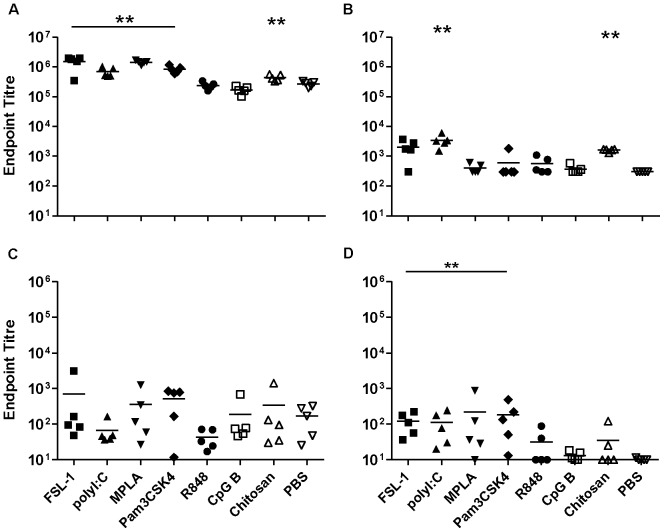
Subcutaneous immunisation with Tetanus toxoid. Endpoint titres for IgG (A, C) and IgA (B, D) in sera (upper panels) and vaginal washes (lower panels) from animals immunised three times with Tetanus toxoid subcutaneously. Asterisks indicate significant differences between the different adjuvant/antigen groups and the PBS control group.

When TT was given subcutaneously, the antigen alone induced very high IgG responses systemically that were enhanced by FSL-1, poly I∶C, MPLA, and Pam_3_CSK_4_ (p<0.01) up to 5.6 fold (Figure 7A). Systemic IgA responses to TT alone were at or below the cut-off for detection (Fig. 7B). Poly I∶C and chitosan induced significant TT specific IgA titres (p<0.01) although modest in comparison to other routes of immunisation. In vaginal wash samples, detectable IgG titres were observed, with no significant differences between groups. Specific IgA responses to TT alone were very low but increased by FSL-1, politic, Pam_3_CSK_4_ (p<0.01) and MPLA (p = 0.04) (Figure 7C and D).

IgG subclass analysis showed that TT given alone induced a very high IgG1/IgG2a ratio, above 50 (Figure S3B). This was significantly reduced by co-administration of TLR agonists: FSL-1, MPLA, Pam_3_CSK_4_, R848 and CpG B.

## Discussion

In the present study, we investigate the impact of a range of TLR ligands as potential adjuvants for different routes of mucosal immunisation and their ability to enhance specific antibody responses to gp140 and TT in systemic and vaginal compartments. In addition we characterize the different impact of TLR adjuvants by route of administration on the balance of Th1/Th2 type humoral immune responses.

Sublingual immunisation (SL) with antigen alone, either gp140 or TT induced good specific systemic IgG and IgA responses (Figure 1 and 2). For IgA these were better than those achieved with subcutaneous (SC) immunisation. The observed responses differ significantly from studies focusing on SL-delivery of HIV gp41, where no systemic or mucosal immune response was detected in the absence of adjuvant [19]. These discrepancies may reflect differences in administered dose, however we have also observed a lack of responsiveness to gp41 in the absence of adjuvant when administered SL (data not shown) suggesting that the nature of the antigen, size and hydrophobicity, may influence uptake and/or immune sensing by this route. However in support of our findings with gp140 and TT, other studies have shown immune responsiveness to a range of immunogens in mice delivered by SL-administration in the absence of adjuvant [20]. While a number of candidate adjuvants in this study showed a trend towards enhanced systemic responses by SL-immunisation over antigen alone, this was only significant for Poly I∶C with gp140. The observation that TT administered alone induced good systemic immune responses confirms previous observations [21], and these were higher than specific systemic responses induced by gp140 alone, furthermore the candidate adjuvants FSL-1, poly I∶C, CpG B and chitosan significantly enhanced systemic responses to TT by the sublingual route. None of the candidate adjuvants significantly enhanced mucosal responses to gp140 or TT above that seen with antigen alone that were higher for specific IgA than IgG, although the most consistent mucosal IgA responses to gp140 were seen with FSL-1, Poly I∶C and CpG B. These results are promising in that they show potent immune induction by the SL-route using a range of TLR adjuvants. Nevertheless, initial humans studies using HPV vaccine (Gardasil®) containing VLPs adjuvanted with alum failed to induce significant immune responses in humans when administered by the SL-route [22] despite inducing good SL-responses in mice [23]. These studies underscore the need to determine whether the reported findings in this study are translatable to humans.

Interestingly, SL-MPLA appeared to reduce specific systemic and mucosal antibody titres to both gp140 and TT. The dampening effects of MPLA on induced immune response might be related to the reported induction of immune tolerance within the oral cavity [24], MPLA promoting the tolerogenic properties of oral Langerhans cells via TLR4 stimulation [24]. However these findings are at odds with clinical studies for allergy vaccines where SL-MPLA increased humoral responses to vaccine allergens [25]. These differences may reflect potential differences in TLR4 expression between humans and mice, different sources of MPLA used or the impact of prior sensitization to an allergen increasing immune responsiveness to SL-immunotherapy.

We cannot completely exclude the possibility that the antigen was at least partially swallowed by the animals following SL-immunisation, even though the volume used was kept to a minimum and the animals were kept under deep anaesthesia after the immunisation with their heads placed in ante-flexion for 10 minutes.

In contrast to SL-immunisation, IN-administration of either gp140 or TT alone gave very poor systemic and mucosal antigen-specific responses. This confirms that, in the absence of an adjuvant, this route of immunisation is a poor site for the induction of strong humoral immune responses [26]. However, all adjuvant candidates examined increased gp140 and TT specific systemic and mucosal IgG responses following IN-application, that when analyzed as a group, were higher than those induced by SL-immunisation and comparable or greater than those following subcutaneous (SC) immunisation. Induced systemic and mucosal IgA responses were also far higher than those induced by adjuvanted SC-immunisation and equivalent or better to those induced by SL-immunisation. Of the individual adjuvant candidates CpG-B appeared to be most effective adjuvant by IN-administration when compared to either SL or SC routes. MPLA also enhanced specific systemic and mucosal responses by the IN-route suggesting the dampening effects observed following SL-administration are likely route specific. R848 appeared to be least effective adjuvant for IN-administration likely reflecting the differences in TLR7/8 expression between mice and humans. In humans TLR7 is mainly expressed in B cells while, in mice, TLR7 is expressed in macrophages, monocytes and dendritic cells [27],[28]. Furthermore TLR8 appears to be non-functional in mice thus, in this animal model, R848 can only work through TLR7 signalling [29].

The vaginal route of administration was the least successful mucosal route for immunisation, with no detectable antibody responses (systemic or mucosal) to gp140 alone or in combination with adjuvants. These findings are in line with other studies using the same antigen in mice [30], Rhesus macaques [17] and humans [22], but at odds with earlier findings in rabbits [31]. These data suggest the rabbit model may be significantly more sensitive to induction of humoral immune responses by this route. The poor inductive potential observed in this study reflects previous studies in mice indicating that the vaginal mucosa is generally considered a poor inductive site for humoral immune responses [32],[33],[34], lacking local organized lymphoid tissue. To determine whether the observed lack of responsiveness was specific to gp140 a smaller pilot study was performed using TT. In contrast to gp140, vaginal immunisation with TT induced significant systemic IgG responses in the absence or presence of adjuvant and detectable mucosal responses were induced when adjuvanted by FSL-1 or Poly I∶C, although responses were still lower than those observed by other mucosal routes of immunisation. It is unclear why vaginal immunisation should be more responsive to TT, but in this respect reflects responses to vaginal infection or replicating vectors [8]. Furthermore, TT was more immunogenic than gp140 across all routes of immunisation.

SC-immunisation induced the most robust systemic IgG responses to gp140 and TT when used alone in comparison to other routes of immunisation. For gp140 these were significantly enhanced both systemically and mucosally when delivered with Pam_3_CSK_4_, while systemic TT responses were enhanced by FSL-1, poly I∶C, MPLA and Pam_3_CSK_4_. Interestingly CpG-B appeared to provide no benefit to SC-immunisation with either gp140 or TT despite having the strongest adjuvant effects on IN-immunisation. These data further indicate that the adjuvant potential of different TLR agonists is influenced by the route of administration. SC-immunisation was notably poor in comparison to SL- or IN-routes with respect to induction of systemic and mucosal IgA responses to gp140 and TT. The observation that SL- and IN-routes of immunisation proved much better than SC-immunisation with respect to specific IgA induction, both systemic and mucosal, is in agreement with previous studies [35].

IgG subclass analysis to address potential Th1/Th2 biasing of immune responses identified some interesting findings. Antigens alone (gp140 and TT) induced different responses according to the route of administration. Both gp140 and TT, gave very high IgG1/IgG2a ratios (>50) with SC-administration indicating a strong Th2 bias. For gp140 this bias was less with SL- (11) and least for IN- (3.5) administration. In contrast, for TT, IN moderately reduced the Th2 bias of SC-immunisation, while SL-administration provided a balance Th1/Th2 ratio (0.9). The strong Th2-type bias of SC-immunisation is supported by previous studies using OVA [36]. Low antigen doses are thought to preferentially stimulate Th2 type responses with Th1 responses more dependent upon antigen reaching draining lymph nodes. Previous studies have shown that SC-administered proteins mostly stay at the site of injection with only minimal amounts reaching draining lymph nodes [37]. It is interesting to speculate that IN- and SL- administration maybe more efficient at delivering antigen to their closely associated lymphoid tissue than SC-immunisation thereby eliciting stronger Th1 responses. This merits further study.

When looking across routes of administration some distinct patters can be recognized. Chitosan appeared to provide a strong Th2 biasing effect for SL- and IN-administration with both TT and gp140. Chitosan is thought to open epithelial tight junctions allowing more efficient uptake of antigen, but may also complex to antigen through electrostactic interactions [15],[38],[39]. This complexing of antigen may restrict access to draining lymph nodes preferentially favouring Th2 type IgG1 dominated responses. In contrast CpG-B reduced the natural Th2 biasing of responses to both antigens irrespective of the route of administration. Different patterns are recognizable when looking at responsiveness by route of administration. For SC-immunisation with gp140 all adjuvants except chitosan reduced the strong Th2 biasing of humoral responses, most clearly demonstrated with MPLA that induced a stronger Th1 bias (Figure 6E). This most likely reflects triggering of antigen loaded dendritic cell maturation and migration to draining lymph nodes along CCL19/CCL21 chemotactic gradients thereby efficiently delivering antigen to a more Th1 type inductive site [36]. This trend was less clear for TT where Poly I∶C, R848 and CpG-B all provided a more balanced Th1/Th2 response but FSL-1, MPLA and Pam_3_CSK_4_ had little or no impact on the strong Th2 bias of TT alone (Figure 7E). SL-immunisation with gp140 was generally Th2 biased, although less so than SC, and only CpG-B and FSL-1 produced an appreciable reduction in IgG1/IgG2a ratios. This may reflect differential TLR expression on localized dendritic cell populations. In contrast SL-immunisation with TT gave a much more balanced Th1/Th2 response with or without adjuvants, the exception being chitosan. IN-immunisation with gp140 provided a more balanced Th1/Th2 profile than SL- or SC-routes, however responses were appreciably shifted towards a Th2 bias by FSL-1, MPLA, Pam_3_CSK_4_ and chitosan. IN-immunisation with TT alone was more skewed toward a Th2 profile (20) than SL-administration with TT and greater than that seen with IN-gp140. A more balanced Th1/Th2 response was induced with Poly I∶C, R848 and CpG-B.

These data demonstrate route, antigen, and adjuvant dependent effects. It is important to take into account that the Balb/C mouse strain, used in our experiments, has a strong Th2 bias compared to other strains of mice [40],[41] and this might explain the high Th2 bias we observed for some experimental groups. Nevertheless the trend for modulating responses toward Th1 or Th2 phenotypes is likely to hold true in other mice species irrespective of their pre-existing bias. Interestingly there was no correlation between the ratio of IgG1/IgG2a and the extent of mucosal IgA responses observed. This suggests that the route of immunisation may play a more important role in the induction of IgA responses than the Th1/Th2 bias of individual TLR ligands.

When taken together our data indicate that IN-immunisation provided the best balance between systemic and mucosal responses with most of the adjuvants evaluated (with notable exceptions discussed above): for systemic and mucosal IgG, IN- was equivalent to SC- and better than SL-immunisation; while for systemic and mucosal IgA, IN- was equivalent to or better than SL- and both were appreciably better than SC-immunisation. Thus IN-immunisation induced comparable systemic and mucosal IgG responses to SC but appreciably better systemic and mucosal IgA responses. Further studies in other species, especially non-human primates and human clinical studies, are needed to assess whether similar results can be confirmed.

## Supporting Information

Figure S1 Serum IgG1/IgG2a ratios after sublingual immunisations. Specific IgG1/IgG2a ratios in sera after three SL immunisations with gp140 (A) or with Tetanus Toxoid (B). Asterisks indicate significant differences between the different adjuvant/antigen groups and the PBS control group.
(TIF)

Figure S2 Serum IgG1/IgG2a ratios after nasal immunisations. Specific IgG1/IgG2a ratios in sera after three IN immunisations with gp140 (A) or with Tetanus Toxoid (B). Asterisks indicate significant differences between the different adjuvant/antigen groups and the PBS control group.
(TIF)

Figure S3 Serum IgG1/IgG2a ratios after subcutaneous immunisations. Specific IgG1/IgG2a ratios in sera after three SC immunisations with gp140 (A) or with Tetanus Toxoid (B). Asterisks indicate significant differences between the different adjuvant/antigen groups and the PBS control group.
(TIF)
